# Distinct Seasonal Patterns of Bacterioplankton Abundance and Dominance of Phyla α-*Proteobacteria* and *Cyanobacteria* in Qinhuangdao Coastal Waters Off the Bohai Sea

**DOI:** 10.3389/fmicb.2017.01579

**Published:** 2017-08-18

**Authors:** Yaodong He, Biswarup Sen, Shuangyan Zhou, Ningdong Xie, Yongfeng Zhang, Jianle Zhang, Guangyi Wang

**Affiliations:** ^1^Center for Marine Environmental Ecology, School of Environmental Science and Engineering, Tianjin University Tianjin, China; ^2^Qinhuangdao Marine Environmental Monitoring Central Station, State Oceanic Administration Qinhuangdao, China; ^3^Key Laboratory of Systems Bioengineering (Ministry of Education), Tianjin University Tianjin, China

**Keywords:** anthropogenic impacts, environmental variables, phylogenetic diversity, bacterioplankton abundance, seasonal variations, redundancy analysis

## Abstract

Qinhuangdao coastal waters in northern China are heavily impacted by anthropogenic and natural activities, and we anticipate a direct influence of the impact on the bacterioplankton abundance and diversity inhabiting the adjacent coastal areas. To ascertain the anthropogenic influences, we first evaluated the seasonal abundance patterns and diversity of bacterioplankton in the coastal areas with varied levels of natural and anthropogenic activities and then analyzed the environmental factors which influenced the abundance patterns. Results indicated distinct patterns in bacterioplankton abundance across the warm and cold seasons in all stations. Total bacterial abundance in the stations ranged from 8.67 × 10^4^ to 2.08 × 10^6^ cells/mL and had significant (*p* < 0.01) positive correlation with total phosphorus (TP), which indicated TP as the key monitoring parameter for anthropogenic impact on nutrients cycling. *Proteobacteria* and *Cyanobacteria* were the most abundant phyla in the Qinhuangdao coastal waters. Redundancy analysis revealed significant (*p* < 0.01) influence of temperature, dissolved oxygen and chlorophyll *a* on the spatiotemporal abundance pattern of α*-Proteobacteria and Cyanobacteria groups.* Among the 19 identified bacterioplankton subgroups, α*-Proteobacteria* (phylum *Proteobacteria*) was the dominant one followed by *Family II* (phylum *Cyanobacteria*), representing 19.1–55.2% and 2.3–54.2% of total sequences, respectively. An inverse relationship (*r* = -0.82) was observed between the two dominant subgroups, α*-Proteobacteria and Family II*. A wide range of inverse Simpson index (10.2 to 105) revealed spatial heterogeneity of bacterioplankton diversity likely resulting from the varied anthropogenic and natural influences. Overall, our results suggested that seasonal variations impose substantial influence on shaping bacterioplankton abundance patterns. In addition, the predominance of only a few cosmopolitan species in the Qinhuangdao coastal wasters was probably an indication of their competitive advantage over other bacterioplankton groups in the degradation of anthropogenic inputs. The results provided an evidence of their ecological significance in coastal waters impacted by seasonal inputs of the natural and anthropogenic matter. In conclusion, the findings anticipate future development of effective indicators of coastal health monitoring and subsequent management strategies to control the anthropogenic inputs in the Qinhuangdao coastal waters.

## Introduction

Marine and coastal environments are of major concern nowadays on account of the rapid developments and growth in anthropogenic activities. Most commonly the anthropogenic activities include terrestrial pollution, aquaculture, urban development, tourism, maritime transport, agricultural and industrial activities, oil refineries, and mining, etc. (Islam and Tanaka, 2004). The nutrient inputs from these activities are the major drivers of eutrophication in most of the coastal areas, ultimately affecting the marine food web components (Vaquer-Sunyer et al., 2015). In addition, rivers and allochthonous biological processes also enrich coastal water with dissolved compounds (Hiriart-Baer et al., 2008; Barrera-Alba et al., 2009; Vargas et al., 2011; Gustafsson et al., 2014; Lafon et al., 2014). Thus, organic matter mineralization and nutrient cycling are crucial for maintenance of coastal ecosystem health. In this context, the assemblage and abundance of bacterioplankton involved in the marine microbial loop (Farooq and Francesca, 2007) are canonical indicators of ecosystem status and the extent of anthropogenic impact on the coastal waters (Zhang et al., 2014). The spatial and temporal dynamics of bacterioplankton community is driven by multiple environmental factors such as latitudinal gradient (Fuhrman et al., 2008; Milici et al., 2016), temperature (Kim et al., 2016), predation (Oguz et al., 2013; Karus et al., 2014), nutrients (Kim et al., 2016), succession (Chen H. et al., 2016), seasons (Gilbert et al., 2012), and timescales (Fuhrman et al., 2015). Apart from natural environmental factors, anthropogenic activities also shape the bacterioplankton assemblage and abundance pattern (Zhang et al., 2009; Thiyagarajan et al., 2010; Zhou et al., 2011; Sauret et al., 2012; Jeffries et al., 2016; Meziti et al., 2016). For instance, the abundances of the bacterioplankton groups that increased and decreased in the impacted sites were significantly correlated with nutrients enrichment (Fodelianakis et al., 2014). Also, there is a direct evidence of specific metal (Cd)-induced patterns in bacterioplankton communities in coastal systems (Wang et al., 2015). In oligotrophic coastal water microcosm with nitrate perturbation, the bacterioplankton community composition was greatly influenced by nitrate loading mode, indicative of nitrate loading impact on marine environments (Dong et al., 2017). Thus, nutrients and toxic pollutants enrichment seem to be the important drivers of the bacterioplankton composition in sites receiving direct human impact (Aguilo-Ferretjans et al., 2008).

A closer inspection of the bacterioplankton community dynamics reveals many interesting relationships and patterns in the taxonomic diversity and abundance. A high concentration of *Synechococcus* and low concentration of *Prochlorococcus* has been observed in sites impacted by human activities with inputs from the land (Bouvy et al., 2012). In a microcosm study, it was found that anthropogenic impacts do not necessarily influence the abundance pattern of rare species (<0.1% of relative abundance), and in fact, nutrients enrichment increased the relative abundance of only abundant species (>1% of relative abundance) *Polaribacter, Tenacibaculum*, and *Rhodobacteraceae* (Baltar et al., 2015). By the use of 16S rRNA gene cloning and sequencing, the bacterioplankton diversity of oligotrophic marine environments is relatively well studied than coastal environments (Zhou et al., 2011; Ortega-Retuerta et al., 2014; Hartmann et al., 2016; Milici et al., 2016; Dong et al., 2017). Bacterioplankton diversity and abundance patterns on a regional-scale and also for local sites under anthropogenic impact remains a hot topic and several reports are available (Simonato et al., 2010; Niu et al., 2011; Peierls and Paerl, 2011; Zhou et al., 2011; Fodelianakis et al., 2014; Laas et al., 2014; Almutairi, 2015; Baltar et al., 2015; Meziti et al., 2015; Xiong et al., 2015; Jeffries et al., 2016). Overall, the available reports on bacterioplankton community composition at regional-scale or in local sites indicate diverse patterns and complex relationships between species composition and environmental factors. In addition, phylogenetic analysis and abundance patterns of bacterioplankton assemblages provide significant insights into the bacterioplankton dynamics in the impacted coastal areas which further leads us to the development of coastal environmental monitoring and management strategies (Demarcq et al., 2012). This calls for more research to identify the bacterioplankton species that show strong linkages with the implicit environmental factors for developing effective descriptors of the anthropogenic impacts.

Qinhuangdao is a famous port city, an important energy export port, and coastal tourist spot on the northwest coast of the Bohai Sea in North China with an area of approximately 7812 km^2^ and a population of 2.9 × 10^6^ (Gu et al., 2016). The Bohai Sea coastline experiences the strong influence of riverine systems, aquaculture, municipal sewage, industrial wastewaters, and agricultural runoffs (Zhu et al., 2014). Land-use surrounding the Qinhuangdao coastal area shifted drastically from agriculture to industrialization in the last 30 years (Xu et al., 2010). The area has undergone development of several petrochemical, steel, and other projects, besides land reclamation and construction of embankments (Zhu et al., 2014). The resulting land-use change has overall influenced the coastal seawater nutrients cycling and associated bacterioplankton, phytoplankton, and other biotic factors (Xu et al., 2010; Liu et al., 2011). Results from other and our previous studies have shown a distinct pattern of bacterioplankton abundance and diversity as a consequence of environmental influence such as estuaries, mariculture, and rainfall (Li J. et al., 2011; Li et al., 2013; Shang et al., 2016; Zhou et al., 2016). These reports further encourage us to investigate closely the relationship between the bacterioplankton community and nutrient levels across different seasons in the near-shore and off-shore stations near Qinhuangdao coast and address the following questions. What are the patterns in abundance of major taxonomic groups and their spatial diversity? What drives the spatiotemporal variations of bacterioplankton in the Qinhuangdao coastal waters?

The objectives of the present study were to: (1) analyze the abundance of total bacteria and major bacterioplankton groups across different seasons, (2) identify the key environmental factors that influence the bacterioplankton abundance, and (3) assess the bacterioplankton distribution and diversity along the near-shore and off-shore coastal areas of Qinhuangdao.

## Materials and Methods

### Study Area and Water Collection

Qinhuangdao, one of the highly urbanized regions, is a port city on the coast of China located in the northeastern Hebei province, and it is situated about 280 km east of Beijing on the northwest Coast of Bohai Sea, the innermost gulf of Yellow Sea. The Qinhuangdao coastal area has a humid continental climate with four distinct seasons. It has a monsoon-influenced humid continental climate with the highest precipitation (152–189 mm) during July–August period. From October month the temperature starts to fall (<5°C) until April, and a very low (<10 mm) precipitation occurs during November to March period (Gu et al., 2016).

**FIGURE 1:**
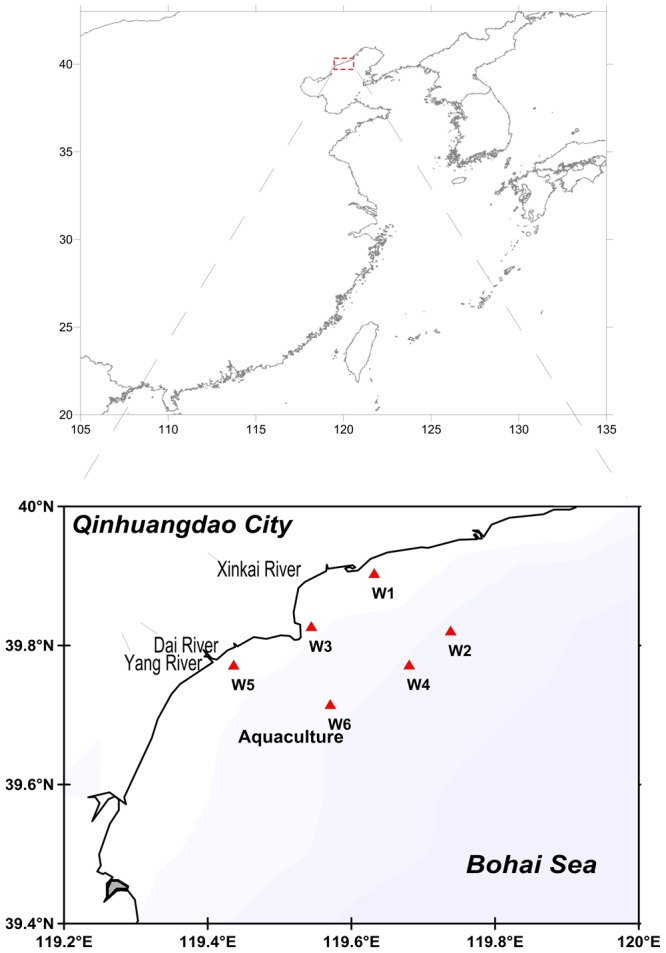
Map of sampling stations.

Six stations (W1–W6) were selected that had the distinct influence of the local environment (**Figure 1**). The W1 station is located outside the port of Qinhuangdao mainly polluted by the shipping industry and municipal sewage, the W3 station is located in the Xinkai river estuary near the Beidaihe Forest Wetland and Qinhuangdao Wildlife Park, and the W5 station is located between the Yang River Estuary and Dai River Estuary. The Yang River and Dai River are severely polluted by microorganisms and the total number of bacteria exceeds the standard levels. W3 and W5 stations are also located in the tourist area and near the bathing beach. W2, W4, and W6 are off-shore stations with aquaculture facility near W6 station. Water samples were collected in the month of March, July, October, and December (2014) at the stations W1, W2, W3, W4, W5, W6 (**Figure 1**). For each month one water sample (1 L) at 1 m depth from the surface was collected using a sample hydrophore. A plexiglass hydrophore (JC-800, Juchuang, China) of capacity 5 L, diameter 15 cm, and height 37.5 cm was used as the sample hydrophore. All samples were stored in sterile glass bottles at 4°C and then transported to the laboratory. A 500 mL volume of water sample was filtered using 0.22 μm membrane filter (AmeriTech Inc., United States). The membrane filters coated with microorganisms were stored at -80°C.

### Water Quality and Nutrient Analysis

The pH, temperature, dissolved oxygen (DO), and salinity of the water samples at six stations were measured using a portable YSI Pro Plus Multiparameter instrument (YSI Inc., United States). The contents of total nitrogen (TN), total phosphorus (TP), ammonia nitrogen (NH_4_-N), nitrate nitrogen (NO_3_-N), nitrite nitrogen (NO_2_-N) and Chlorophyll a (Chl *a*) were determined in water samples in accordance with the “Specification for oceanographic survey” (GB/T 12763.4-2007). Briefly, NH_4_-N was measured by sodium bromate oxidation method which involves oxidization of ammonium salt to nitrite in alkaline medium, followed by determination of the TN content using diazo spectrophotometry. After deducting the concentration of NO_2_-N, the concentration of NH_4_-N was estimated. NO_3_-N was measured by zinc cadmium reduction method where nitrate is quantitatively reduced to nitrite in water by zinc cadmium reduction method following determination of total nitrite by the diazo and azo method, the original nitrite was then corrected and the nitrate content was calculated. NO_2_-N was measured by a diazo azo method which involves the reaction of nitrite with sulfanilamide in acidic medium following product reaction with hydrochloric acid and synthesis of a red azo dye whose absorbance was measured at 543 nm. Chl *a* measurement was done by an extraction-fluorimetric method which involves excitation by blue light of the acetone extract of Chl *a* to produce red fluorescence (Holm-Hansen and Bo, 1978). The fluorescence value of the extract was determined at 685 nm before and after acidification.

The abundance of total bacteria at the 6 stations near Qinhuangdao coastal area over warm and cold seasons were monitored using fluorescence microscopy to assess the influence of anthropogenic and natural activities in the near-shore and off-shore waters. The total bacterial count in water samples (formaldehyde-fixed) at each station was estimated by fluorescence microscopy (Eclipse Ni-U, Nikon Instruments Inc., United States). Sample (2 mL water) filtration was done at a pressure of 150 mm Hg through a polycarbonate filter (0.22 μm aperture, 25 mm diameter) (AmeriTech Inc., United States). The membrane filter was stained with 2X SYBR-Gold solution (300 μl) and was visualized at 480–485 nm wavelength (blue light excitation). Data reported are the means of counts in 20 randomly selected fields per sample. For each station, triplicate samples were processed and for each replicate sample, bacterial numbers were counted in 20 randomly selected fields. The data reported are the means of counts in triplicate samples.

### DNA Isolation and Sequence Analysis of Bacterioplankton Assemblage

Five hundred milliliter of surface water samples collected in July 2014 from 6 stations were filtered onto 0.2 μm polycarbonate membrane filters (AmeriTech Inc., United States). The resulting filters were stored in ultra-low temperature refrigerator (-80°C) until DNA isolation. The total DNA was extracted using E.Z.N.A.^TM^ Water DNA Kit (Omega Bio-Tek Inc., United States) following the manufacturer’s instructions. The isolated DNA was used as template for bacterial 16S rRNA gene amplification using universal primers 27F (5′-AGAGTTTGATCCTGGCTCAG-3′) and 1492R (5′-GGTTACCTTGTTACGACTT-3′) on Bio-Rad S1000 thermal cycler (Bio-Rad Laboratories Inc., United States) using the following protocol: 95°C 5 min; 94°C 45 s, 55°C 45 s, 72°C 1 min; 35 cycles; 72°C 10 min. PCR amplification for each sample was performed in triplicates and the triplicate PCR products were mixed to obtain the final sample for clone library construction. All PCR products were gel-purified, cloned into a pMD18-T Simple Vector (Sino Biological Inc., China), and then transformed into *Escherichia coli* DH5α competent cells by traditional heat shock treatment. 16S rRNA clone libraries of a total of six water samples (one sample per station) for the month of July were constructed and 90 positive clones were randomly picked from each sample plate for sequence analysis (Beijing Genomics Institution, China). The coverage of the clone libraries ranged from 31.1 to 56.7% and the impact of low coverage on diversity analysis was minimized by calculating Simpson diversity estimate (Haegeman et al., 2013) in Mothur software package (Schloss et al., 2009). The sequences have been submitted to NCBI Genebank database with accession number MF498066 – MF498485.

All sequence analyses were performed within versions 1.35.1 of the Mothur software package (Schloss et al., 2009). The FASTA format sequences were aligned with SILVA alignments and the distance was calculated at cutoff 0.03 (97% sequence homology) using *align.seqs* and *dist.seqs* commands, respectively. Chimeras were identified using *chimera.uchime* command with default parameters. Sequences were classified using *classify.seqs* command with reference to Ribosomal Database Project (RDP) as the taxonomy file (silva.bacteria.rdp.tax) and the *remove.lineage* command was used to remove the sequences that belong to mitochondria, chloroplast, eukaryote, archaea, and unknown lineages. Cluster command was used to assign sequences to OTUs with opticlust clustering method. α-diversity was calculated by counting the number of OTUs and using the reciprocal of Simpson Index (invsimpson). The invsimpson calculator is preferred to other measures of α-diversity as it indicates the richness in a community with a uniform evenness that would have the same level of diversity. Moreover, the inverse of the Simpson index has some biological interpretation and do not tend to be affected by sampling effort because it is independent of abundance distributions (Haegeman et al., 2013).

### Quantitative PCR Analysis of Bacterioplankton Groups

To assess the phylum/class level abundance distribution of total bacteria in warm and cold seasons, qPCR experiments with phylum and class specific primers (Supplementary Table S1) were conducted for all 6 stations. qPCR method was used to detect and quantitate the abundance of the major phylogenetic groups of bacterioplankton in the water samples from Qinhuangdao coastal area. The advantage of qPCR method in our study was its ability to quantitate specific bacterioplankton groups by using phylum/class specific primers. In addition, qPCR quantitation is fast, reliable, and accurate. Based on the results of clone library analysis and identification, the following dominant classes were selected to carry out quantitative PCR experiments: α-*Proteobacteria*, β-*Proteobacteria, Cyanobacteria, Actinobacteria, Firmicutes*, and *Bacteroidetes*. PCR was carried out in a 20 μL reaction mixture containing 1 μL DNA template (2.8-10.5 ng/μL), 10 μL SYBR Select Master Mix (Thermo Fisher Scientific Inc.), 1 μL forward primer (10 nmol/mL), 1 μL reverse primer (10 nmol/mL), and 7 μL nuclease-free molecular-grade water. The qPCR cycling condition was: 95°C 55 s; 95°C 20 s, 60°C 35 s, 72°C 30 s; 40 cycles; 95°C 1 min, 60°C 1 min, heating-up from 75 to 95°C, the increment of 0.5°C for 8 s. The annealing temperature of each primer is shown in Supplementary Table S1. The standard curves were constructed with known amounts of plasmid DNA containing the sequence insert. For the positive plasmid preparation, the PCR products were ligated to pMD18-T Vector by pMD18-T Vector Cloning Kit (Takara Biotechnology (Dalian) Co., Ltd.) and then transformed into *E. coli* competent cells (DH5α) followed by a screening of positive clones. Plasmid DNA was extracted using E.Z.N.A^®^ Plasmid Midi Kit (Omega Bio-Tek Inc., United States) and the concentration of the extracted DNA was measured using NanoDrop ND-1000 spectrophotometer (Nanodrop Technology). The gene copies were calculated using the mean mass of pMD18-T Vector ligated with the target DNA sequence. DNA of each sample and standard plasmid DNA dilution (10^1^ and 10^5^) were used as templates for qPCR. Each sample and standard were analyzed in triplicates. The standard curve was constructed to estimate the copy number of each sample according to the dilution ratio and copy number of the standard plasmid template.

### Statistical Analysis

Principal component analysis (PCA) was done to reveal the relationships between the environmental parameters and the distribution of the sampling stations in an ordination plot. Pearson’s correlation analysis was performed to assess the correlation between the abundance of total bacteria, major bacterioplankton groups, and environmental parameters. Redundancy analysis (RDA) was carried out to identify the explanatory environmental parameters that significantly (*p* < 0.01) constrain the bacterioplankton abundance data by forward selection in Canoco version 5.02. RDA was performed using abundance (qPCR data) and environmental data. The PCA of environmental parameters was done using normalized (*z*-score) data in Canoco version 5.02. For RDA, the qPCR data were log(x+1) transformed and the environmental data was normalized by *z*-score.

## Results

### High Seasonal Variations of Environmental Parameters

The environmental parameters across 6 stations near Qinhuangdao coastal area appeared highly heterogeneous spatiotemporally. In the warm season (July–October), the temperature of coastal waters in all stations was within 21–25°C, whereas in the cold season (December–March) the temperature was significantly lower ranging from -0.3 to 6.8°C. pH was notably higher (11.6 ± 0.09) in October at all stations. Salinity levels were similar (∼29 ppt) in all stations and also during warm and cold seasons. The DO levels were relatively higher in cold period than warm as a result of temperature effect on oxygen solubility in water. The average values of environmental parameters (nutrients) with their variation over different periods at the 6 stations near Qinhuangdao coastal area are shown in **Table 1**. The NH_4_-N level was high in W5 station than that of the others. In October the NH_4_-N was relatively high at all stations (0.05–0.17 mg/L). No significant variation in NO_2_-N and NO_3_-N content was observed at the 6 stations. TN was detected in all stations (0.35–0.6 mg/L) with relatively large fluctuations (0.6–1.8mg/L) in the warm season (July) and was lowest in the cold season (December). In July and March, the TP was found to be comparatively higher than in October and December. Furthermore, the average TP content was higher in W1, W2, W3, W5 (0.12–0.13 mg/L) than that in W4 and W6 stations (0.07–0.08 mg/L). W1 and W5 stations showed highest average Chl *a* level (11.6–16.0 mg/m^3^) followed by W3 and W6 (5.6–5.9 mg/m^3^) and lowest levels at W2 and W4 (2.6-3.2 mg/m^3^). The variation in Chl *a* level was substantially less in W2 and W4 stations suggesting a low abundance of phytoplankton at these stations than at the polluted stations (W1, W3, W5, and W6). In the Bohai Sea, silicates and Si/dissolved inorganic N ratio have been associated with phytoplankton abundance (Chen Y.H. et al., 2016). In addition, climate and mariculture activity have a significant correlation with the Chl *a* trends (Fu et al., 2016). River discharge and suspended sediment also influence Chl *a* in Bohai Sea coast. These factors might have played a role in the spatial variation of Chl *a* observed in the present study. Also, Chl *a* mostly was higher in July in all stations probably due to more growth of phytoplankton resulting from optimal light and temperature conditions.

**Table 1:** The data of environmental parameters at near-shore (W1, W3, and W5) and off-shore (W2, W4, and W6) stations near Qinhuangdao coastal area.

Stations	NH_4_-N (mg/L)	NO_2_-N _(_mg/L)	NO_3_-N (mg/L)	TN (mg/L)	TP (mg/L)	Chl *a* (mg/m^3^)
W1	0.067 (0.037)	0.035 (0.024)	0.337 (0.196)	0.793 (0.199)	0.131 (0.080)	11.604 (6.334)
W3	0.061 (0.032)	0.024 (0.017)	0.355 (0.419)	1.103 (0.542)	0.126 (0.077)	5.603 (5.389)
W5	0.104 (0.048)	0.028 (0.018)	0.337 (0.248)	0.959 (0.447)	0.128 (0.019)	16.004 (10.559)
W2	0.053 (0.012)	0.024 (0.017)	0.435 (0.431)	1.158 (0.506)	0.121 (0.079)	3.209 (1.181)
W4	0.041 (0.015)	0.024 (0.022)	0.362 (0.296)	0.851 (0.334)	0.079 (0.054)	2.651 (1.826)
W6	0.054 (0.017)	0.024 (0.019)	0.331 (0.189)	0.937 (0.461)	0.069 (0.063)	5.873 (8.444)

*TN, TP, and Chl *a* indicate total nitrogen, total phosphate, and Chlorophyll a.*

*Mean (*SD*) of values over 4 seasons (July, October, December, and March) are provided.*

**FIGURE 2:**
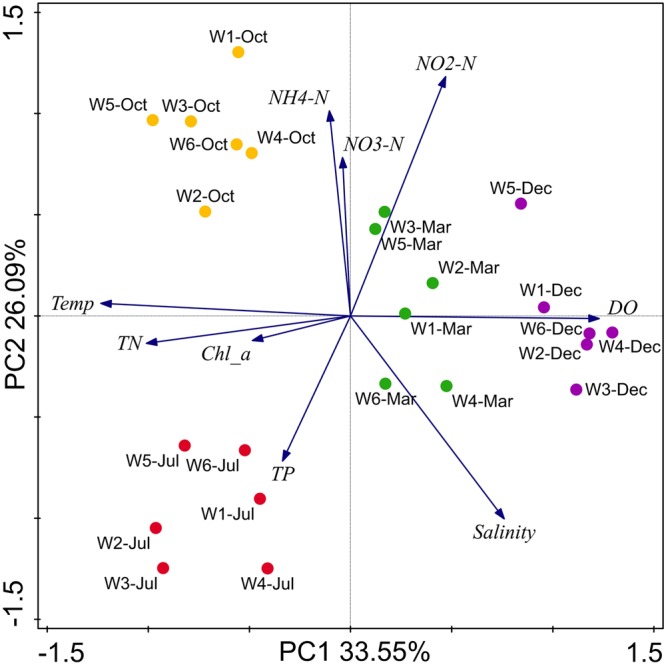
Biplot of the principal component analysis (PCA) for environmental parameters in the sampling stations near Qinhuangdao coastal area. The two principal components (PC1 and PC2) explained 59.64% of total variation in environmental data, and shows clear partitioning of July and October samples from December and March along the PC1, and partitioning of July from October along PC2. PC1 and PC2 strongly correlated to variables NH_4_-N and NO_3_-N, and total nitrogen (TN), temperature (T) and Chlorophyll-*a* (Chl *a*), respectively. Total phosphate (TP), NO_2_-N, and salinity were not correlated to PC1 and PC2. Blue arrows represent environmental parameters, and circles in color represents sampling points.

By analyzing all the measured environmental variables in combination, the resultant ordination plot showed distinct partitioning of coastal water samples across warm and cold seasons rather than across near-shore and off-shore stations (**Figure 2**). The ordination plot could explain 59.6% of total variation in the environmental data and revealed a linear positive correlation between TN, temperature, and Chl *a*, and a negative correlation of DO with these variables. These parameters were most crucial in the partitioning of samples from the warm and cold seasons. Similarly, NH_4_-N and NO_3_-N had a positive correlation with each other, and salinity, NO_2_-N, and TP did not show any notable correlations to any of the other variables. Overall, the PCA biplot clearly showed that seasonal variation of the measured environmental parameters was more than spatial variation. Thus, seasonal loads of anthropogenic inputs seem to have a significant impact on the nutrients conditions in the coastal areas of Qinhuangdao.

### Bacterioplankton Abundance Patterns and Influencing Factors

**FIGURE 3:**
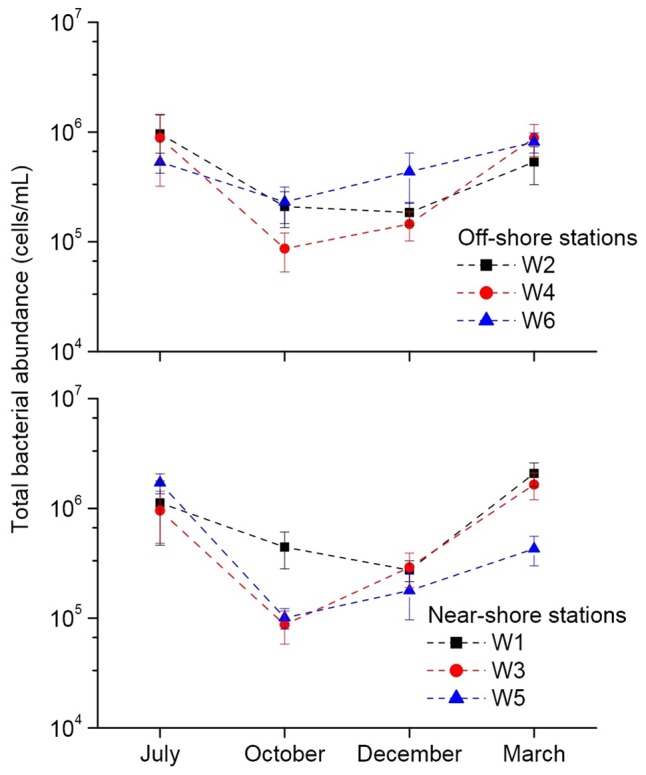
Fluorescence microscopy based quantification of total bacteria at the near-shore and off-shore stations near the Qinhuangdao coastal area of Bohai Sea. ‘W1’ to ‘W6’ indicate the different sampling stations.

**FIGURE 4:**
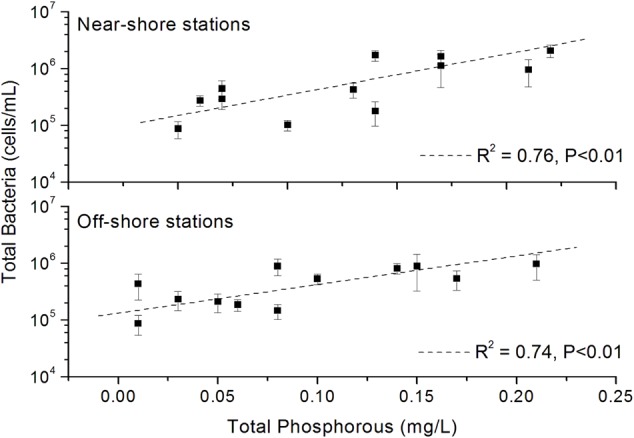
Significant (*p* < 0.01) correlation between total bacterial abundance and total phosphorous. Values on *Y*-axis are shown in log-scale.

The results of fluorescence microscopy based detection of total bacterial abundance in the near-shore and off-shore stations are shown in **Figure 3**. In July, all the stations mostly showed highest abundance (5.32 × 10^5^ – 1.71 × 10^6^ cells/mL) of bacteria compared to other months. Overall, the total bacterial abundance in each of the 6 stations over the 4 months was in between 8.67 × 10^4^ and 2.08 × 10^6^ cells/mL. Within the other 3 months, March recorded higher abundance than October and December in all the stations. The stations W1, W3, W5, and W6 typically had relatively more abundance than the W2 and W4 perhaps due to nutrients overload in these stations as a consequence of higher anthropogenic and natural activities as evident from their higher level of Chl *a*. A significant (*p* < 0.01) positive correlation of total bacterial abundance with TP was seen (**Figure 4**).

**FIGURE 5:**
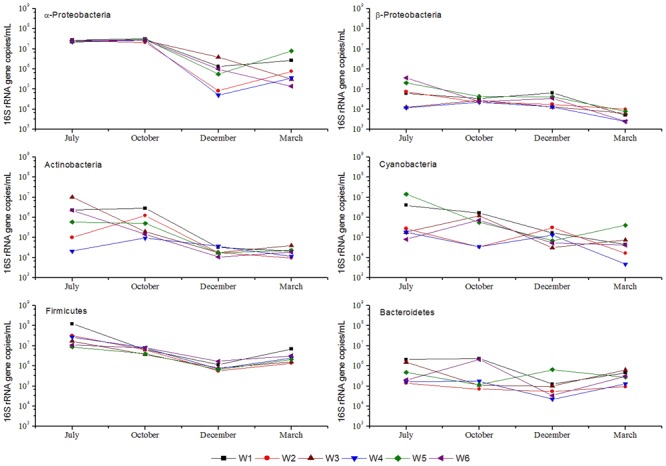
16S rRNA gene abundance (qPCR quantification) of major phylogenetic groups from coastal bacterioplankton assemblages in 6 stations near Qinhuangdao coastal area. The symbols and lines in black, red, green, blue, cyan, and magenta represent stations W1, W2, W3, W4, W5, and W6. Values on *Y*-axis are plotted in log-scale.

The qPCR based abundance estimates of 6 major phylum/class i.e., α-*Proteobacteria*, β-*Proteobacteria, Cyanobacteria, Actinobacteria, Firmicutes*, and *Bacteroidetes* are shown in **Figure 5**. α-*Proteobacteria, Actinobacteria, Firmicutes*, and *Bacteroidetes* showed a fairly similar pattern of abundance distribution in the 6 stations. Notably, in the warm season, these 4 groups exhibited relatively higher abundances than in cold season. However, β-*Proteobacteria* and *Cyanobacteria* showed distinct trends, wherein the former one showed a similar pattern in all stations over the 4 months, i.e., higher abundance in cold than warm season. *Bacteroidetes* abundance at W1 and W6 stations remained high in October, and in W5 its abundance increased only in July and December. Near-shore and off-shore differences in the abundance of the major phylotypes were also apparent. In the warm season, near-shore stations possessed more *Cyanobacteria* and *Bacteroidetes* than off-shore stations. Alternatively, in cold months, near-shore stations possessed a relatively higher abundance of α-*Proteobacteria, Firmicutes, Actinobacteria*, and β-*Proteobacteria* than off-shore stations.

**FIGURE 6:**
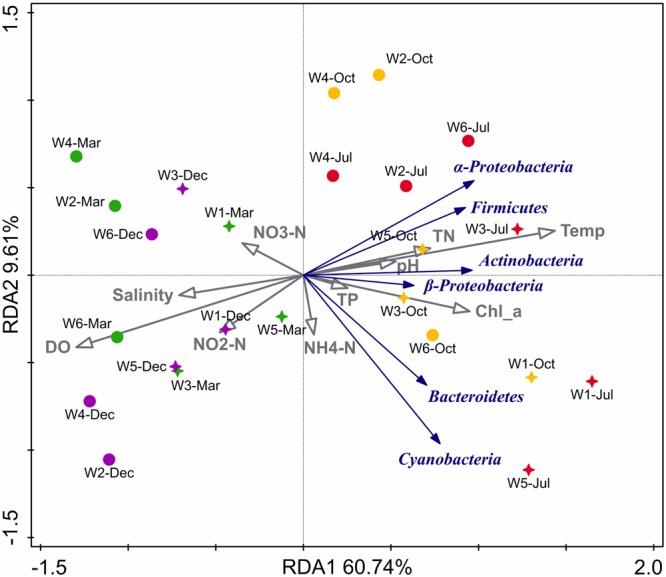
Biplot of redundancy analysis (RDA) showing relationship between the environmental variables and 16S rRNA gene abundance (qPCR quantification) of major phylogenetic groups in six stations near Qinhuangdao coastal area. The two RDA axes (RDA1 and RDA2) explained 70.35% of total variation in abundance data, and the significant (*p* < 0.01) explanatory variables were temperature (temp), dissolved oxygen (DO), and Chlorophyll a (Chl_*a*). Blue arrows represent different phylotypes, gray arrows represent environmental parameters, stars represent near-shore samples and circles represent off-shore samples. Different colors of the star or circle symbols represent different sampling time, with March samples in green, July samples in red, October samples in yellow and December samples in purple.

**Table 2** shows the Pearson’s correlation coefficients for the relationship between bacterioplankton abundance and some environmental parameters. α-*Proteobacteria* showed significant (*p* < 0.05) positive correlation with TN, Chl *a*, DO, and temperature. Furthermore, from the ordination plot (**Figure 6**) based on redundancy analysis, temperature and Chl *a* were the major factors that positively correlated with the first principal component and were the significant explanatory parameters (*p* < 0.01) that constrained the abundance of α-*Proteobacteria* and *Cyanobacteria* phylotypes. On the contrary, DO showed a negative influence (*p* < 0.01) and salinity was non-significant in explaining any variation of the abundance of bacterioplankton groups. Overall, samples collected in warm July or October had a much higher abundance of α-*Proteobacteria, Firmicutes*, and *Actinobacteria* than that of cold March or December. In contrast, β-*Proteobacteria, Cyanobacteria*, and *Bacteroidetes* did not show any distinct seasonal influence which likely suggests their diverse metabolic potential and ability to utilize organic matter even at low concentrations that allow them to attain invariable growth throughout the year.

**Table 2:** Pearson’s correlation analysis showing correlations between abundance of total bacteria, bacterioplankton groups and some environmental parameters.

Bacterial groups	Total nitrogen (mg/L)	Total phosphate (mg/L)	Chlorophyll-*a* (mg/m^3^)	Dissolved oxygen (mg/L)	Temperature (°C)
Total bacteria	NS	0.719^∗∗^(0.00)	NS	NS	NS
α-*Proteobacteria*	0.490^∗^(0.015)	NS	0.461^∗^(0.023)	NS	0.974^∗∗^(0.00)
β-*Proteobacteria*	NS	NS	0.532^∗∗^(0.007)	NS	NS
*Cyanobacteria*	NS	NS	NS	NS	NS
*Actinobacteria*	NS	NS	NS	NS	NS
*Bacteroidetes*	NS	NS	NS	NS	0.410^∗^(0.046)
*Firmicutes*	NS	NS	NS	NS	NS

*^∗∗^*P* < 0.01, ^∗^*P* < 0.05; NS, non-significant.*

*The values represents correlation coefficient (significant bilateral) for sample number *N* = 24. Total bacteria and bacterioplankton group abundance data were based on fluorescence microscopy and quantitative PCR, respectively.*

### Spatial Distribution and Diversity of Bacterioplankton Assemblages

**FIGURE 7:**
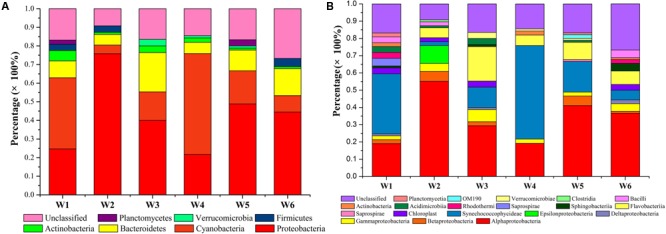
Composition and distribution of bacterioplankton assemblages at the 6 stations near Qinhuangdao coastal area. Percentage distribution of the 16S rRNA gene sequences at the **(A)** phylum level and **(B)** class level indicates higher relative abundance of subgroups *Proteobacteria, Cyanobacteria, Bacteroidetes*, and *Actinobacteria*.

The assemblage of bacterioplankton communities featuring diversity and distribution at the 6 stations in the warm season was assessed and the results are reported in **Table 3** and **Figure 7**. Based on the environmental data, July exhibited greater levels and fluctuations in the measured parameters perhaps due to more nutrient inputs that resulted from anthropogenic and natural activities in warm season. Thus, further characterization of bacterioplankton phylogeny, composition, and diversity was conducted on water samples collected in July. Out of the selected clones analyzed, 62, 62, 68, 44, 47, and 71 OTUs were identified in W1, W2, W3, W4, W5, and W6 stations, respectively (**Table 3**). A broad phylogenetic distribution of the bacterioplankton community at the 6 stations in warm season is shown in **Figure 7A**. The analysis of 16S rRNA gene sequences from these stations revealed 7 distinct phyla (**Figure 7A**). The majority of the sequences generated from the 6 bacterioplankton clone libraries were affiliated to phyla *Proteobacteria* (21.7–75.9%), *Cyanobacteria* (4.6–54.2%), *Bacteroidetes* (9–21.2%), *Actinobacteria* (1.1–5.6%), *Firmicutes* (0–4.4%), *Verrucomicrobia* (0–3.5%), *Planctomycetes* (0–3.3%). The unclassified group represented sequences in the range of 9.2–26.7%. At the phylum level *Proteobacteria (49% of total sequences), Cyanobacteria (24% of total sequences)*, and *Bacteroidetes (17% of total sequences)* were the dominant groups in all the stations. There were 19 bacterioplankton classes in the total sequences acquired from the coastal waters of Qinhuangdao (**Figure 7B**). The predominant phylum *Proteobacteria* comprised of five classes namely α-*Proteobacteria*, γ-*Proteobacteria*, 𝜀-*Proteobacteria*, β-*Proteobacteria*, and δ-*Proteobacteria* accounting to ca. 82, 9, 3.6, 2, and 1.6% of *Proteobacteria*, respectively. Further taxonomic resolution of the assemblages revealed nearly 30 different families that were assigned to the identified OTUs from the bacterioplankton assemblages (Supplementary Figure S1). The OTUs within *Proteobacteria* phylum were assigned to 15 families: *Campylobacteraceae, Comamonadaceae, Methylophilaceae, Pseudobacteriovoracaceae, Burkholderiaceae, Bacteriovoracaceae, Moraxellaceae, Halomonadaceae, Oceanospirillaceae, Enterobacteriaceae, Rhodospirillaceae*, SAR11, *Hyphomicrobiaceae, Rhodobiaceae*, and *Rhodobacteraceae*. The second major phylum *Cyanobacteria* comprised OTUs which were all assigned to a single family *— Family II*. The OTUs representing phylum *Bacteroidetes* belonged to 4 families: *Flavobacteriaceae, Cryomorphaceae, Saprospiraceae*, and *Chitinophagaceae*. *Rhodobacteraceae* represented major proportion (>10% of total 16S r RNA sequences) in all the stations followed by *Family* II especially in W1 and W4 stations. Only three OTUs assigned to *Rhodobacteraceae, Family II*, and *Cryomorphaceae* were cosmopolitans, i.e., they were found in all the stations. Overall, α-*Proteobacteria (Rhodobacteraceae)* (19.1–55.2%) and *Cyanobacteria* (*Family II)* (2.3–54.2%) were the most dominant cosmopolitan groups. Interestingly, it was noted that these two dominant groups had an inverse relationship (*r* = -0.82).

**Table 3:** The number of 16S rRNA sequences, operational taxonomic units (OTUs), and inverse Simpson diversity (1/D) estimates for the bacterioplankton clone libraries.

Stations	16S rRNA Sequence number	Operational taxonomic units (OTUs)	Inverse Simpson diversity index (1/D)
W1	87	62	34.9
W2	85	62	83.0
W3	80	68	105.0
W4	82	44	16.3
W5	85	47	10.2
W6	89	71	70.3

*W1–W6 represents the 6 stations near Qinhuangdao coastal area for which bacterioplankton clone libraries were constructed and analyzed.*

Based on inverse Simpson index (1/D), the ascending order of diversity at the 6 stations was W5 < W4 < W1 < W6 < W2 < W3 ranging from 10.2 to 105 (**Table 3**). The results suggested a wide difference in the bacterioplankton diversity, which likely resulted from the presumed shifts in the environmental conditions of the coastal waters of Qinhuangdao. The inverse Simpson index (1/D) may serve as a sensitive indicator of the anthropogenic impact in the Bohai Sea and can accurately monitor the extent of human activities in Qinhuangdao coastal region.

## Discussion

### Spatiotemporal Variations and Factors Influencing Bacterioplankton Abundance

Most global-scale studies on the biogeography of bacterioplankton communities have revealed intriguing findings and have largely shown that diversity and abundance pattern are closely linked to latitudinal gradient (Fuhrman et al., 2008), seasons (Cuevas et al., 2004; Fuhrman et al., 2015), temperature and nutrient levels (Pommier et al., 2007; Vichi et al., 2007). In addition, previous studies have well established that bacterioplankton abundance is potentially linked to elevated anthropogenic nutrient loads in aquatic ecosystems. Overall, nutrient contents (TP, TN, Chl *a*, and DO) and gradient of the natural parameter (temperature) displayed significant differences across seasons and sampling stations in our study. In the warm season, we observed a greater concentration of nutrients (TN, TP, and Chl *a*) and decreased DO which is consistent with other studies (Lee et al., 2011; Shang et al., 2016). The heavy rainfall in the warm season seems one of the main causes which overload organic matter and pollutants through the estuaries and run-offs. Freshwater inflows and aquaculture activities also deliver nutrients and contaminants besides nutrients from fertilizers and anthropogenic activities, which may also cause the nutrient concentration variations, and shape the bacterioplankton abundance pattern (Jeffries et al., 2016). Generally, low salinity in coastal water indicates that the area has received river discharge, however, in the present study any significant lowering of salinity in the coastal waters was not observed. This perhaps is an indication that Qinhuangdao coastal area does not experience the heavy impact of riverine system round the year. In addition to nutrient inputs, the direct input of pathogenic and fastidious microorganisms from wastewater treatment, wildlife sanctuary, and sewage sources, which are known to influence the bacterioplankton abundance (Vandewalle et al., 2012; Liu et al., 2015) also might affect bacterioplankton abundance and diversity in Qinhuangdao coastal waters.

Our results demonstrated that bacterioplankton abundance in the Qinhuangdao coastal waters exhibit substantial shifts over time and relatively less in space. Furthermore, the strong correlation in total phosphorous and abundance agree well with previous findings that describe inorganic phosphorous as an essential nutrient and that it serves a vital role in cellular energy storage and transformation and is also a growth-limiting nutrient (Brandsma, 2016). Although bacterioplankton and phytoplankton abundance (Chl *a*) in many studies are closely linked, our study show no relationship between them, an uncoupling phenomenon often observed when inorganic phosphorus is available (Robarts and Carr, 2009). The considerable reduction in bacterioplankton abundance in October perhaps is an indication of anthropogenic inputs or short-term physical forcing events that bring in nutrients which offer phytoplankton an opportunity to outgrow bacterioplankton. The higher abundance of bacterioplankton in warm July and cold March is probably associated with dissolved organic carbon of algal exudates from algal bloom and allochthonous carbon, respectively. Overall, this study extends upon the mechanism of bacterioplankton dynamics and explains the consistency of abundance pattern with the utilization of specific substrates. Nevertheless, the tight connection of abundance with TP bespeaks high implication in coastal pollution monitoring.

Bacterioplankton abundance in the near-shore area is larger than that in the open ocean, especially in the area of the Gulf and the estuarine area. In our study, generally the near-shore abundance was higher than off-shore, perhaps caused by the uneven distribution of organic matter in the coastal stations that are affected directly by run-offs, sewage, human activities, etc. than off-shore stations. As the seawater gets polluted by industrial wastewater and/or domestic sewage, the bacterioplankton may go in a state of unstable nutrition which subsequently affects the total bacterioplankton abundance, transiently. In our study, the abundance was relatively higher in the stations adjacent to river estuaries than that of other stations, while within the near-shore stations, the difference was not significant. Interestingly, in station W5 the bacterioplankton abundance fluctuated dramatically that possibly indicated a strong influence of nearby river estuaries heavily polluted with invasive microorganisms. The results showed that the total abundance in the stations near Qinhuangdao coastal area fluctuated between 8.67 × 10^4^ (W1 in March) to 2.08 × 10^6^ (W4 in October) cells/ml which is in good agreement with the estimate projected by Whitman et al. (Whitman et al., 1998) and empirical data (Al-Rifaie et al., 2008; Robarts and Carr, 2009; Li H.-Y. et al., 2011).

Although total abundance pattern provides an indication of anthropogenic impacts on coastal waters to a certain extent, knowing the pattern of variation in the composition of bacterioplankton allows further understanding of how the growth of different groups of bacterioplankton is controlled by sporadic nutrient inputs. In addition, different phylogenetic groups of bacterioplankton play a specialized role in their consumption of nutrients, and thus dissecting their abundance fluctuations would provide greater insight into the extent of nutrient loading and their recycling by functional groups. The distinct pattern of seasonal variations in the abundance of α-*Proteobacteria, Actinobacteria, Firmicutes*, and *Bacteroidetes* groups most likely explains their role in organic matter transformation as chemoheterotrophs. These groups exhibited higher abundance mostly in warm season when there were intense anthropogenic activities and subsequent enrichment of seawater with pollutants. In fact, it was shown that warming promotes bacterioplankton to benefit from the phytoplankton bloom and increase their abundance ultimately shifting toward a more heterotrophic system (Scheibner et al., 2014). These groups perhaps are directly involved in the decomposition of anthropogenic chemicals and algal exudates via *a* microbial loop as evident from their strong relationship with TN and Chl *a*. In contrast, β-*Proteobacteria* was found to be tightly coupled with Chl *a*, which probably indicated that this less known bacterioplankton group feeds mostly on the algal exudates and may not be directly involved in the decomposition of complex polymers. *Bacteroidetes* also take part in organic matter degradation and have been associated with the occurrence of algal bloom. Some members of this group use proteorhodopsin to capture light energy for growth making them photoheterotrophic (Gomez-Consarnau et al., 2007). The strong correlation between *Bacteroidetes* group and temperature could likely be a direct upshot of more light intensity in the warm season.

In our study, temperature, TN, Chl *a*, and DO were identified as key drivers of spatiotemporal dissimilarity between bacterioplankton abundance patterns in the coastal waters of Qinhuangdao area. Salinity, temperature, and pH are naturally varying parameters, while nutrient concentrations and DO levels indicate the extent of anthropogenic activities besides allochthonous inputs. Temperature and salinity are strong drivers of variations in microbial assemblages of estuaries (Jeffries et al., 2016), but in coastal water salinity changes are marginal, and therefore non-significant in explaining community variations. Previous studies have reported strong association of TN, Chl *a*, temperature, and TP with the microbial community composition variations and perhaps are the major drivers of community dynamics in aquatic ecosystems (Fodelianakis et al., 2014; Wang et al., 2015; Jeffries et al., 2016; Dong et al., 2017). However, in the present study, these key parameters were tenuous in partitioning the near-shore and off-shore waters near Qinhuangdao area, perhaps indicating the influence of other factors such as Si/dissolved inorganic N ratio, river discharge, short-term physical forcing events, and suspended sediments. In conclusion, this study shows temporal variations in the environmental parameters were more pronounced than spatial in shaping the abundance patterns of bacterioplankton inhabiting the coastal waters of Qinhuangdao. This further connotes the consequence of sporadic anthropogenic activities and their chemical inputs in influencing the bacterioplankton assemblages in the harbor area. Our study provided the first insight into the bacterioplankton ecology of an anthropogenically impacted regional-scale coastal water of Qinhuangdao, by combining bacterioplankton abundance patterns with physicochemical characteristics.

### Spatial Patterns of Bacterioplankton Composition and Diversity

The regional and global ocean sampling expeditions have highlighted the importance of bacterioplankton community dynamics and their assembly into functional communities by elucidating their community structure inhabiting different marine locations (Yutin et al., 2007; Lauro et al., 2014). Yet, the information on bacterioplankton diversity within littoral areas adjoining harbors is quite limited perhaps due to complex interactions of multitudinous factors that underline their structure and distribution. As far as the littoral area of Bohai Sea is concerned, this is one of the first studies to our knowledge which reports the diversity of the bacterioplankton assemblage nearby harbor and the factors that shape their structure and composition. *Proteobacteria, Cyanobacteria, Actinobacteria*, and *Bacteroidetes* represented the major phylotypes at the 6 stations, and is in agreement with other reports which have found these bacterioplankton groups to be the major inhabitant of marine habitats (Li J. et al., 2011; Li et al., 2013; Smedile et al., 2014; Xiong et al., 2015; Jeffries et al., 2016). *Proteobacteria* and *Cyanobacteria* exhibited higher dynamics in their composition among others. Although it is quite challenging to relate phylogenetic diversity and ecological function in marine microbial diversity, yet it is hypothesized that these major groups efficiently metabolize exudates and dissolved organic matter composed of carbohydrates and/or amino acids derived from both growing and senescent phytoplankton (Teira et al., 2008). In addition, these groups are also known to degrade hydrocarbons and inorganic compounds from allochthonous sources (Robarts and Carr, 2009).

Within phylum *Proteobacteria*, the class α-*Proteobacteria* represented the majority of the 16S rRNA gene sequences and was the predominant group in all stations. α-proteobacteria are usually abundant in coastal waters and they can form the predominant surface- and particle-colonizing group (Dang and Lovell, 2002a,b). *Rhodobacteraceae* (also called the marine *Roseobacter* clade) is usually the largest subgroup in the α-proteobacteria and they are the pioneering and dominant microorganisms on submerged surfaces and organic particles (Dang and Lovell, 2000; Dang and Lovell, 2016). The class γ-*Proteobacteria* constituted only a minor fraction in our molecular-based study, although members of this class are best known for their rapid growth and also represented as a major group in global surveys (Pommier et al., 2007). OTUs assigned to α-*Proteobacteria* and *Cyanobacteria* phyla were cosmopolitans in our study, and this finding is consistent with previous reports (Pommier et al., 2007; Smedile et al., 2014). *Cyanobacteria* dominated at W1 and W4 stations and most OTUs belonged to the genus *Synechococcus*. They are mainly free-living and are the main participants and contributors to primary productivity of the global carbon cycle and are the common bacteria inhabiting coastal, temperate, and cold environments (Brown et al., 2014; Xia et al., 2015). From genome analysis, *Synechococcus* sp. WH8102 was found to be able to use some new organic compounds as sources of nitrogen and phosphorus (Palenik et al., 2003). In addition, *Cyanobacteria* (*Synechococcus*) are typically more abundant in higher nutrients or dynamic conditions and are often described as generalist better suited to exploit the fluctuating environments (Palenik et al., 2003). In contrast, *Rhodobacteraceae* comprise mainly aerobic photo- and chemo-heterotrophs besides purple non-sulfur bacteria which perform photosynthesis in anaerobic environments. Members of this subgroup are metabolically heterogeneous and are capable of anoxygenic phototrophy, aromatic and organosulfur degradation (Lenk et al., 2012). They are the main groups involved in the demethylation of dimethylsulfoniopropionate in the water column (Curson et al., 2011). They are largely involved in sulfur and carbon biogeochemical cycling and symbiosis with other micro- and macro-organisms (Pujalte et al., 2014). The dominance of *Rhodobacteraceae* in the coastal waters of Qinhuangdao port area epitomizes the degradation of organic and inorganic compounds containing sulfur. The inverse relationship between the two most predominant taxonomic groups (*Rhodobacteraceae* and *Family II*) may be either due to competition, niche partitioning, or another mechanism. Further investigation is needed to ascertain the exact cause of the inverse relationship. The low proportion of γ- and δ-*Proteobacteria* at the 6 stations perhaps indicate the absence of chronic polyaromatic hydrocarbons (PAHs) influence in the coastal waters near Qinhuangdao port, because these groups are often seen associated with high levels of chronic PAHs (Jeanbille et al., 2016). Moreover, the SAR11 considered as highly diverse and abundant at different depths and across habitat types within euphotic and mesopelagic zones was detected only in few stations (W1, W2, and W5). The absence of SAR11 clade at other stations perhaps indicates their rarity and thus this clade was unable to represent in our clone libraries. Nevertheless, the use of inverse Simpson Index which does not tend to be affected by rare species could predict diversity more accurately than other estimators of diversity, and thus diversity analysis in our study was relatively robust and could give a reliable and meaningful interpretation. The wide range and high variation in diversity estimate (1/D) indicate environmental heterogeneity of the Qinhuangdao coastal waters due to varied nutrient types and their concentrations that directly influence the relative abundance of bacterioplankton functional groups.

In the present study, the richness (species number) of the bacterioplankton community was not estimated taking into account the high rate of estimation error with low coverage libraries (Haegeman et al., 2013). Instead, we implemented the estimation of Simpson diversity index which is a robust estimator and provides an accurate estimation of the species diversity. It is independent of any species abundance distributions (SADs) unlike the richness estimators (Haegeman et al., 2013). The use of Simpson index in our study assured accurate estimation of bacterioplankton diversity even with low coverage clone libraries. Also, as explained in an earlier study, coverage does not correlate with library size and the authors clearly mentioned that “a large library size did not guarantee a stable estimate of phylotype richness, nor was a small library necessarily too small” (Kemp and Aller, 2004). The authors also showed that the average size of 220 bacterial libraries was 81 ± 10, ranging in size from 5 to 417 clones. Therefore, our study with 90 clones per library is in good agreement with the average size 81 ± 10 (Kemp and Aller, 2004).

Overall, our study on the bacterioplankton diversity at stations near Qinhuangdao port suggests prominence of only two cosmopolitan OTUs with several endemic OTUs. The predominance of few species is probably an indication of selective advantage of these species to overcome a load of chemical inputs despite scatting grazing and predation by virus and macro-organisms. Also, the effect of anthropogenic and allochthonous inputs was evident from the wide range of inverse Simpson diversity estimates for bacterioplankton assemblages at the stations near Qinhuangdao port. This is consistent with the notion that disturbed marine and coastal environments possess high variations of diversity as a result of complex and unstable nutrient resources, in contrary to oligotrophic marine habitats and deep oceans. The overall findings of this study are expected to serve the basis for Qinhuangdao coastal water monitoring and development of strategies for anthropogenically impacted coastal areas.

## Author Contributions

YH has contributed to data acquisition, analysis and drafting the work. BS has contributed to data interpretation, analysis, drafting the work and critical revision. SZ, YZ, and JZ have contributed to data acquisition. NX helped in data analysis. GW contributed to conception of the work, data interpretation and manuscript revision. All authors have final approval to the published version and accountable for all aspects of the work.

## Conflict of Interest Statement

The authors declare that the research was conducted in the absence of any commercial or financial relationships that could be construed as a potential conflict of interest.

## References

[B1] Aguilo-Ferretjans M. M. Bosch R. Martin-Cardona C. Lalucat J. Nogales B. (2008). Phylogenetic analysis of the composition of bacterial communities in human-exploited coastal environments from Mallorca Island (Spain). *Syst. Appl. Microbiol.* 31 231–240. 10.1016/j.syapm.2008.04.003 18572341

[B2] Almutairi A. (2015). Spatial–temporal variations and diversity of the bacterioplankton communities in the coastal waters of Kuwait. *Mar. Pollut. Bull.* 100 699–709. 10.1016/j.marpolbul.2015.09.016 26404068

[B3] Al-Rifaie K. Al-Yamani F. Polikarpov I. (2008). First study of the bacterioplankton distribution in the Northwestern Arabian Gulf. *Mar. Ecol. J.* 574 43–48.

[B4] Baltar F. Palovaara J. Vila-Costa M. Salazar G. Calvo E. Pelejero C. (2015). Response of rare, common and abundant bacterioplankton to anthropogenic perturbations in a Mediterranean coastal site. *FEMS Microbiol. Ecol.* 91:fiv058 10.1093/femsec/fiv058 26032602

[B5] Barrera-Alba J. J. Gianesella S. M. F. Moser G. A. O. Saldanha-Corrêa F. M. P. (2009). Influence of allochthonous organic matter on bacterioplankton biomass and activity in a eutrophic, sub-tropical estuary. *Estuar. Coast. Shelf Sci.* 82 84–94. 10.1016/j.ecss.2008.12.020

[B6] Bouvy M. Dupuy C. Pagano M. Barani A. Charpy L. (2012). Do human activities affect the picoplankton structure of the Ahe atoll lagoon (Tuamotu Archipelago, French Polynesia)? *Mar. Pollut. Bull.* 65 516–524. 10.1016/j.marpolbul.2012.01.008 22306310

[B7] Brandsma J. (2016). Phytoplankton phenotype plasticity induced by phosphorus starvation may play a significant role in marine microbial ecology and biogeochemistry. *New Phytol.* 211 765–766. 10.1111/nph.14085 27397523

[B8] Brown M. V. Ostrowski M. Grzymski J. J. Lauro F. M. (2014). A trait based perspective on the biogeography of common and abundant marine bacterioplankton clades. *Mar. Genomics* 15 17–28. 10.1016/j.margen.2014.03.002 24662471

[B9] Chen H. Zhang H. Xiong J. Wang K. Zhu J. Zhu X. (2016). Successional trajectories of bacterioplankton community over the complete cycle of a sudden phytoplankton bloom in the Xiangshan Bay, East China Sea. *Environ. Pollut.* 219 750–759. 10.1016/j.envpol.2016.07.035 27453358

[B10] Chen Y. H. Gao Y. H. Chen C. P. Liang J. R. Sun L. Zhen Y. (2016). Seasonal variations of phytoplankton assemblages and its relation to environmental variables in a scallop culture sea area of Bohai Bay, China. *Mar. Pollut. Bull.* 113 362–370. 10.1016/j.marpolbul.2016.10.025 27771098

[B11] Cuevas L. A. Daneri G. Jacob B. Montero P. (2004). Microbial abundance and activity in the seasonal upwelling area off Concepción (36°S), central Chile: a comparison of upwelling and non-upwelling conditions. *Deep Sea Res. Part II* 51 2427–2440. 10.1016/j.dsr2.2004.07.026

[B12] Curson A. R. J. Todd J. D. Sullivan M. J. Johnston A. W. B. (2011). Catabolism of dimethylsulphoniopropionate: microorganisms, enzymes and genes. *Nat. Rev. Microbiol.* 9 849–859. 10.1038/nrmicro2653 21986900

[B13] Dang H. Lovell C. R. (2000). Bacterial primary colonization and early succession on surfaces in marine waters as determined by amplified rRNA gene restriction analysis and sequence analysis of 16S rRNA genes. *Appl. Environ. Microbiol.* 66 467–475. 10.1128/AEM.66.2.467-475.2000 10653705 PMC91850

[B14] Dang H. Lovell C. R. (2002a). Numerical dominance and phylotype diversity of marine rhodobacter species during early colonization of submerged surfaces in coastal marine waters as determined by 16S ribosomal DNA sequence analysis and fluorescence In Situ hybridization. *Appl. Environ. Microbiol.* 68 496–504. 11823183 10.1128/AEM.68.2.496-504.2002 PMC126732

[B15] Dang H. Lovell C. R. (2002b). Seasonal dynamics of particle-associated and free-living marine *Proteobacteria* in a salt marsh tidal creek as determined using fluorescence in situ hybridization. *Environ. Microbiol.* 4 287–295. 12030854 10.1046/j.1462-2920.2002.00295.x

[B16] Dang H. Lovell C. R. (2016). Microbial surface colonization and biofilm development in marine environments. *Microbiol. Mol. Biol. Rev.* 80 91–138. 10.1128/MMBR.00037-15 26700108 PMC4711185

[B17] Demarcq H. Reygondeau G. Alvain S. Vantrepotte V. (2012). Monitoring marine phytoplankton seasonality from space. *Remote Sens. Environ.* 117 211–222. 10.1016/j.rse.2011.09.019

[B18] Dong Z. Wang K. Chen X. Zhu J. Hu C. Zhang D. (2017). Temporal dynamics of bacterioplankton communities in response to excessive nitrate loading in oligotrophic coastal water. *Mar. Pollut. Bull.* 114 656–663. 10.1016/j.marpolbul.2016.10.041 27773533

[B19] Farooq A. Francesca M. (2007). Microbial structuring of marine ecosystems. *Nat. Rev. Microbiol.* 5 782–791. 10.1038/nrmicro1747 17853906

[B20] Fodelianakis S. Papageorgiou N. Pitta P. Kasapidis P. Karakassis I. Ladoukakis E. D. (2014). The pattern of change in the abundances of specific bacterioplankton groups is consistent across different nutrient-enriched habitats in Crete. *Appl. Environ. Microbiol.* 80 3784–3792. 10.1128/aem.00088-14 24747897 PMC4054211

[B21] Fu Y. Xu S. Liu J. (2016). Temporal-spatial variations and developing trends of Chlorophyll-a in the Bohai Sea, China. *Estuar. Coast. Shelf Sci.* 173 49–56. 10.1016/j.ecss.2016.02.016

[B22] Fuhrman J. A. Cram J. A. Needham D. M. (2015). Marine microbial community dynamics and their ecological interpretation. *Nat. Rev. Microbiol.* 13 133–146. 10.1038/nrmicro3417 25659323

[B23] Fuhrman J. A. Steele J. A. Hewson I. Schwalbach M. S. Brown M. V. Green J. L. (2008). A latitudinal diversity gradient in planktonic marine bacteria. *Proc. Natl. Acad. Sci. U.S.A.* 105 7774–7778. 10.1073/pnas.0803070105 18509059 PMC2409396

[B24] Gilbert J. A. Steele J. A. Caporaso J. G. Steinbruck L. Reeder J. Temperton B. (2012). Defining seasonal marine microbial community dynamics. *ISME J.* 6 298–308. 10.1038/ismej.2011.107 21850055 PMC3260500

[B25] Gomez-Consarnau L. Gonzalez J. M. Coll-Llado M. Gourdon P. Pascher T. Neutze R. (2007). Light stimulates growth of proteorhodopsin-containing marine Flavobacteria. *Nature* 445 210–213. 10.1038/nature05381 17215843

[B26] Gu J. Hu C.-F. Kuang C.-P. Kolditz O. Shao H.-B. Zhang J.-B. (2016). A water quality model applied for the rivers into the Qinhuangdao coastal water in the Bohai Sea, China. *J. Hydrodynam. Ser. B* 28 905–913. 10.1016/S1001-6058(16)60691-1

[B27] Gustafsson E. Deutsch B. Gustafsson B. G. Humborg C. Mörth C. M. (2014). Carbon cycling in the Baltic Sea — The fate of allochthonous organic carbon and its impact on air–sea CO2 exchange. *J. Mar. Syst.* 129 289–302. 10.1016/j.jmarsys.2013.07.005

[B28] Haegeman B. Hamelin J. Moriarty J. Neal P. Dushoff J. Weitz J. S. (2013). Robust estimation of microbial diversity in theory and in practice. *ISME J.* 7 1092–1101. 10.1038/ismej.2013.10 23407313 PMC3660670

[B29] Hartmann M. Hill P. G. Tynan E. Achterberg E. P. Leakey R. J. Zubkov M. V. (2016). Resilience of SAR11 bacteria to rapid acidification in the high-latitude open ocean. *FEMS Microbiol. Ecol.* 92:fiv161 10.1093/femsec/fiv161 26691595

[B30] Hiriart-Baer V. P. Diep N. Smith R. E. H. (2008). Dissolved organic matter in the great lakes: role and nature of allochthonous material. *J. Great Lakes Res.* 34 383–394. 10.3394/0380-1330

[B31] Holm-Hansen O. Bo R. (1978). Chlorophyll a determination: improvements in methodology. *Oikos* 30 438–447. 10.2307/3543338

[B32] Islam M. S. Tanaka M. (2004). Impacts of pollution on coastal and marine ecosystems including coastal and marine fisheries and approach for management: a review and synthesis. *Mar. Pollut. Bull.* 48 624–649. 10.1016/j.marpolbul.2003.12.004 15041420

[B33] Jeanbille M. Gury J. Duran R. Tronczynski J. Agogué H. Ben Saïd O. (2016). Response of core microbial consortia to chronic hydrocarbon contaminations in coastal sediment habitats. *Front. Microbiol.* 7:1637 10.3389/fmicb.2016.01637 PMC5061854 27790213

[B34] Jeffries T. C. Schmitz Fontes M. L. Harrison D. P. Van-Dongen-Vogels V. Eyre B. D. Ralph P. J. (2016). Bacterioplankton dynamics within a large anthropogenically impacted urban estuary. *Front. Microbiol.* 6:1438 10.3389/fmicb.2015.01438 PMC4726783 26858690

[B35] Karus K. Paaver T. Agasild H. Zingel P. (2014). The effects of predation by planktivorous juvenile fish on the microbial food web. *Eur. J. Protistol.* 50 109–121. 10.1016/j.ejop.2014.01.006 24703613

[B36] Kemp P. F. Aller J. Y. (2004). Bacterial diversity in aquatic and other environments: what 16S rDNA libraries can tell us. *FEMS Microbiol. Ecol.* 47 161–177. 10.1016/s0168-6496(03)00257-5 19712332

[B37] Kim H. J. Jung S. W. Lim D.-I. Jang M.-C. Lee T.-K. Shin K. (2016). Effects of temperature and nutrients on changes in genetic diversity of bacterioplankton communities in a semi-closed bay, South Korea. *Mar. Pollut. Bull.* 106 139–148. 10.1016/j.marpolbul.2016.03.015 27001714

[B38] Laas P. Simm J. Lips I. Metsis M. (2014). Spatial variability of winter bacterioplankton community composition in the Gulf of Finland (the Baltic Sea). *J. Mar. Syst.* 129 127–134. 10.1016/j.jmarsys.2013.07.016

[B39] Lafon A. Silva N. Vargas C. A. (2014). Contribution of allochthonous organic carbon across the serrano river basin and the adjacent fjord system in Southern Chilean Patagonia: insights from the combined use of stable isotope and fatty acid biomarkers. *Prog. Oceanogr.* 129 98–113. 10.1016/j.pocean.2014.03.004

[B40] Lauro F. M. Senstius S. J. Cullen J. Neches R. Jensen R. M. Brown M. V. (2014). The common oceanographer: crowdsourcing the collection of oceanographic data. *PLoS Biol.* 12:e1001947 10.1371/journal.pbio.1001947 PMC4159111 25203659

[B41] Lee S. B. Birch G. F. Lemckert C. J. (2011). Field and modelling investigations of fresh-water plume behaviour in response to infrequent high-precipitation events, Sydney Estuary, Australia. *Estuar. Coast. Shelf Sci.* 92 389–402. 10.1016/j.ecss.2011.01.013

[B42] Lenk S. Moraru C. Hahnke S. Arnds J. Richter M. Kube M. (2012). Roseobacter clade bacteria are abundant in coastal sediments and encode a novel combination of sulfur oxidation genes. *ISME J.* 6 2178–2187. 10.1038/ismej.2012.66 22739490 PMC3504970

[B43] Li H.-Y. Chen M.-X. Li G. Zheng T.-L. Zheng S.-L. Chen B. (2011). Occurrence of total and culturable bacteria in Shenzhen coastal waters and their application in the environment assessment. *Mar. Environ. Sci.* 30 487–491.

[B44] Li J. Li F. Yu S. Qin S. Wang G. (2013). Impacts of mariculture on the diversity of bacterial communities within intertidal sediments in the Northeast of China. *Microb. Ecol.* 66 861–870. 10.1007/s00248-013-0272-6 23963221

[B45] Li J. Wang G. Qin S. (2011). Microbial communities of sediments influenced by mariculture from the coast of Qinhuangdao. *Ecol. Environ. Sci.* 20 920–926.

[B46] Liu S. M. Li L. W. Zhang Z. (2011). Inventory of nutrients in the Bohai. *Cont. Shelf Res.* 31 1790–1797. 10.1016/j.csr.2011.08.004

[B47] Liu Y. Dong Q. Shi H. (2015). Distribution and population structure characteristics of microorganisms in urban sewage system. *Appl. Microbiol. Biotechnol.* 99 7723–7734. 10.1007/s00253-015-6661-7 25981998

[B48] Meziti A. Kormas K. A. Moustaka-Gouni M. Karayanni H. (2015). Spatially uniform but temporally variable bacterioplankton in a semi-enclosed coastal area. *Syst. Appl. Microbiol.* 38 358–367. 10.1016/j.syapm.2015.04.003 25976032

[B49] Meziti A. Tsementzi D. Ar Kormas K. Karayanni H. Konstantinidis K. T. (2016). Anthropogenic effects on bacterial diversity and function along a river-to-estuary gradient in Northwest Greece revealed by metagenomics. *Environ. Microbiol.* 18 4640–4652. 10.1111/1462-2920.13303 27001690

[B50] Milici M. Tomasch J. Wos-Oxley M. L. Wang H. Jauregui R. Camarinha-Silva A. (2016). Low diversity of planktonic bacteria in the tropical ocean. *Sci. Rep.* 6:19054 10.1038/srep19054 PMC4707477 26750451

[B51] Niu Y. Shen H. Chen J. Xie P. Yang X. Tao M. (2011). Phytoplankton community succession shaping bacterioplankton community composition in Lake Taihu, China. *Water Res.* 45 4169–4182. 10.1016/j.watres.2011.05.022 21684570

[B52] Oguz T. Macias D. Renault L. Ruiz J. Tintore J. (2013). Controls of plankton production by pelagic fish predation and resource availability in the Alboran and Balearic Seas. *Prog. Oceanogr.* 11 1–14. 10.1016/j.pocean.2013.03.001

[B53] Ortega-Retuerta E. Fichot C. G. Arrigo K. R. Van Dijken G. L. Joux F. (2014). Response of marine bacterioplankton to a massive under-ice phytoplankton bloom in the Chukchi Sea (Western Arctic Ocean). *Deep Sea Res. Part II* 105 74–84. 10.1016/j.dsr2.2014.03.015

[B54] Palenik B. Brahamsha B. Larimer F. W. Land M. Hauser L. Chain P. (2003). The genome of a motile marine Synechococcus. *Nature* 424 1037–1042. 10.1038/nature01943 12917641

[B55] Peierls B. L. Paerl H. W. (2011). Longitudinal and depth variation of bacterioplankton productivity and related factors in a temperate estuary. *Estuar. Coast. Shelf Sci.* 95 207–215. 10.1016/j.ecss.2011.08.033

[B56] Pommier T. Canback B. Riemann L. Bostrom K. H. Simu K. Lundberg P. (2007). Global patterns of diversity and community structure in marine bacterioplankton. *Mol. Ecol.* 16 867–880. 10.1111/j.1365-294X.2006.03189.x 17284217

[B57] Pujalte M. J. Lucena T. Ruvira M. A. Arahal D. R. Macián M. C. (2014). “The family Rhodobacteraceae,” in *The Prokaryotes: Alphaproteobacteria* and *Betaproteobacteria* eds Rosenberg E. DeLong E. F. Lory S. Stackebrandt E. Thompson F. (Berlin: Springer) 439–512.

[B58] Robarts R. D. Carr G. M. (2009). “Bacteria, Bacterioplankton A2 - likens, gene E,” in *Encyclopedia of Inland Waters* ed. Likens G. E. (Oxford: Academic Press) 193–200. 10.1016/B978-012370626-3.00124-1

[B59] Sauret C. Christaki U. Moutsaki P. Hatzianestis I. Gogou A. Ghiglione J.-F. (2012). Influence of pollution history on the response of coastal bacterial and nanoeukaryote communities to crude oil and biostimulation assays. *Mar. Environ. Res.* 79 70–78. 10.1016/j.marenvres.2012.05.006 22743577

[B60] Scheibner M. V. Dörge P. Biermann A. Sommer U. Hoppe H.-G. Jürgens K. (2014). Impact of warming on phyto-bacterioplankton coupling and bacterial community composition in experimental mesocosms. *Environ. Microbiol.* 16 718–733. 10.1111/1462-2920.12195 23869806

[B61] Schloss P. D. Westcott S. L. Ryabin T. Hall J. R. Hartmann M. Hollister E. B. (2009). Introducing mothur: open-source, platform-independent, community-supported software for describing and comparing microbial communities. *Appl. Environ. Microbiol.* 75 7537–7541. 10.1128/aem.01541-09 19801464 PMC2786419

[B62] Shang J.-Y. Zhou S.-Y. Wang Z.-H. Li Z.-B. Xie N.-D. Ma W.-M. (2016). Effects of rainfall on the total number of bacteria and the composition of culturable bacteria in Qinhuangdao West Beach. *Microbiology* 43 1228–1234.

[B63] Simonato F. Gómez-Pereira P. R. Fuchs B. M. Amann R. (2010). Bacterioplankton diversity and community composition in the Southern Lagoon of Venice. *Syst. Appl. Microbiol.* 33 128–138. 10.1016/j.syapm.2009.12.006 20227843

[B64] Smedile F. Messina E. La Cono V. Yakimov M. M. (2014). Comparative analysis of deep-sea bacterioplankton OMICS revealed the occurrence of habitat-specific genomic attributes. *Mar. Genomics* 17 1–8. 10.1016/j.margen.2014.06.001 24937756

[B65] Teira E. Gasol J. M. Aranguren-Gassis M. Fernández A. González J. Lekunberri I. (2008). Linkages between bacterioplankton community composition, heterotrophic carbon cycling and environmental conditions in a highly dynamic coastal ecosystem. *Environ. Microbiol.* 10 906–917. 10.1111/j.1462-2920.2007.01509.x 18215158

[B66] Thiyagarajan V. Tsoi M. M. Y. Zhang W. Qian P. Y. (2010). Temporal variation of coastal surface sediment bacterial communities along an environmental pollution gradient. *Mar. Environ. Res.* 70 56–64. 10.1016/j.marenvres.2010.03.002 20359741

[B67] Vandewalle J. L. Goetz G. W. Huse S. M. Morrison H. G. Sogin M. L. Hoffmann R. G. (2012). Acinetobacter, Aeromonas and Trichococcus populations dominate the microbial community within urban sewer infrastructure. *Environ. Microbiol.* 14 2538–2552. 10.1111/j.1462-2920.2012.02757.x 22524675 PMC3427404

[B68] Vaquer-Sunyer R. Conley D. J. Muthusamy S. Lindh M. V. Pinhassi J. Kritzberg E. S. (2015). Dissolved organic nitrogen inputs from wastewater treatment plant effluents increase responses of planktonic metabolic rates to warming. *Environ. Sci. Technol.* 49 11411–11420. 10.1021/acs.est.5b00674 26356812

[B69] Vargas C. A. Martinez R. A. San Martin V. Aguayo M. Silva N. Torres R. (2011). Allochthonous subsidies of organic matter across a lake–river–fjord landscape in the Chilean Patagonia: implications for marine zooplankton in inner fjord areas. *Cont. Shelf Res.* 31 187–201. 10.1016/j.csr.2010.06.016

[B70] Vichi M. Masina S. Navarra A. (2007). A generalized model of pelagic biogeochemistry for the global ocean ecosystem. Part II: numerical simulations. *J. Mar. Syst.* 64 110–134. 10.1016/j.jmarsys.2006.03.014

[B71] Wang K. Zhang D. Xiong J. Chen X. Zheng J. Hu C. (2015). Response of bacterioplankton communities to cadmium exposure in coastal water microcosms with high temporal variability. *Appl. Environ. Microbiol.* 81 231–240. 10.1128/aem.02562-14 25326310 PMC4272717

[B72] Whitman W. B. Coleman D. C. Wiebe W. J. (1998). Prokaryotes: the unseen majority. *Proc. Natl. Acad. Sci. U.S.A.* 95 6578–6583. 10.1073/pnas.95.12.6578 9618454 PMC33863

[B73] Xia X. Guo W. Liu H. (2015). Dynamics of the bacterial and archaeal communities in the Northern South China Sea revealed by 454 pyrosequencing of the 16S rRNA gene. *Deep Sea Res. Part II* 117 97–107. 10.1016/j.dsr2.2015.05.016

[B74] Xiong J. Chen H. Hu C. Ye X. Kong D. Zhang D. (2015). Evidence of bacterioplankton community adaptation in response to long-term mariculture disturbance. *Sci. Rep.* 5:15274 10.1038/srep15274 PMC4607939 26471739

[B75] Xu S. Song J. Li X. Yuan H. Li N. Duan L. (2010). Changes in nitrogen and phosphorus and their effects on phytoplankton in the Bohai Sea. *Chin. J. Oceanol. Limnol.* 28 945–952. 10.1007/s00343-010-0005-3

[B76] Yutin N. Suzuki M. T. Teeling H. Weber M. Venter J. C. Rusch D. B. (2007). Assessing diversity and biogeography of aerobic anoxygenic phototrophic bacteria in surface waters of the Atlantic and Pacific Oceans using the global ocean sampling expedition metagenomes. *Environ. Microbiol.* 9 1464–1475. 10.1111/j.1462-2920.2007.01265.x 17504484

[B77] Zhang D. Wang X. Xiong J. Zhu J. Wang Y. Zhao Q. (2014). Bacterioplankton assemblages as biological indicators of shrimp health status. *Ecol. Indic.* 38 218–224. 10.1016/j.ecolind.2013.11.002

[B78] Zhang R. Lau S. C. Ki J. S. Thiyagarajan V. Qian P. Y. (2009). Response of bacterioplankton community structures to hydrological conditions and anthropogenic pollution in contrasting subtropical environments. *FEMS Microbiol. Ecol.* 69 449–460. 10.1111/j.1574-6941.2009.00726.x 19619230

[B79] Zhou S.-Y. Chen X.-N. Cui L. Song Z.-Q. Li Z.-B. Zhang S. (2016). Impacts of environmental factors on bacterial diversity of Xinkai river estuary in the coastal area of Qinhuangdao. *Microbiology* 43 2578–2593.

[B80] Zhou W. Long A. Jiang T. Chen S. Huang L. Huang H. (2011). Bacterioplanktondynamics along the gradient from highly eutrophic Pearl River Estuary to oligotrophic northern South China Sea in wet season: implication for anthropogenic inputs. *Mar. Pollut. Bull.* 62 726–733. 10.1016/j.marpolbul.2011.01.018 21316714

[B81] Zhu L. Wu J. Xu Z. Xu Y. Lin J. Hu R. (2014). Coastline movement and change along the Bohai Sea from 1987 to 2012. *J. Appl. Remote Sens.* 8:083585 10.1117/1.JRS.8.083585

