# Supplementary material for: Distinct Seasonal Patterns of Bacterioplankton Abundance and Dominance of Phyla α-Proteobacteria and Cyanobacteria in Qinhuangdao Coastal Waters Off the Bohai Sea
Source: Front Microbiol. 2017 Aug 18;8:1579. doi: 10.3389/fmicb.2017.01579 (PMC5563310; doi:10.3389/fmicb.2017.01579)
Supplement: Supplementary file 1 [file Table_1.docx]

Supplementary Material

**Distinct seasonal patterns of bacterioplankton abundance and dominance of phyla α-Proteobacteria and Cyanobacteria in Qinhuangdao coastal waters off the Bohai Sea**

**Yaodong He^1, †^, Biswarup Sen^1, †^, Shuangyan Zhou^1^, Ningdong Xie^1^, Yongfeng Zhang^2^, Jianle Zhang^2^, Guangyi Wang^1, 3, *^**

**^†^**These authors have contributed equally to this work.

**^*^Correspondence:** Corresponding Author: [gywang@tju.edu.cn](mailto:gywang@tju.edu.cn)

**Supplementary Table S1- List of primers used in qPCR experiments**

| Phylum | Primer name | Primer sequence  (5ʹ→3ʹ) | Annealing temperature (°C) | Reference |
| --- | --- | --- | --- | --- |
| *α-Proteobacteria* | Eub338F | ACTCCTACGGGAGGCAGCAG | 60.0 | Lane, 1991 |
|  | Alf685R | TCTACGRATTTCACCYCTAC | 60.0 | Overmann et al., 1999 |
| *β-Proteobacteria* | Eub338F | ACTCCTACGGGAGGCAGCAG | 60.0 | Lane, 1991 |
|  | Bet680R | TCACTGCTACACGYG | 60.0 | Stach et al., 2003 |
| *Cyanobacteria* | Cya106F | CGGACGGGTGAGTAACGCGTGA | 60.0 | Nübel et al., 1997 |
|  | Cya359R | CCCATTGCGGAARATTCCCC | 60.0 | Nübel et al., 1997 |
| *Actinobacteria* | Act235F | CGCGGCCTATCAGCTTGTTG | 60.0 | Manz et al., 1996 |
|  | Eub518R | ATTACCGCGGCTGCTGG | 60.0 | Muyzer et al., 1993 |
| *Bacteroidetes* | Bac798F | CRAACAGGATTAGATACCCT | 61.5 | Bacchetti De Gregoris et al., 2011 |
|  | Bac967R | GGTAAGGTTCCTCGCGTAT | 61.5 | Bacchetti De Gregoris et al., 2011 |
| *Firmicutes* | Fir928F | TGAAACTYAAAGGAATTGACG | 61.5 | Bacchetti De Gregoris et al., 2011 |
|  | Fir1040R | ACCATGCACCACCTGTC | 61.5 | Bacchetti De Gregoris et al., 2011 |

**References**

Lane, D. J. (1991). 16S/23S rRNA sequencing. Nucleic acid techniques in bacterial systematics. E. Stackebrandt and M. Goodfellow, eds. New York, NY, John Wiley and Sons: 115-175.

Overmann, J., Coolen, M.J.L., and Tuschak, C. (1999). Specific detection of different phylogenetic groups of chemocline bacteria based on PCR and denaturing gradient gel electrophoresis of 16S rRNA gene fragments. *Arch. Microbiol.* 172(2), 83-94. doi: 10.1007/s002030050744.

Stach, J.E.M., Maldonado, L.A., Ward, A.C., Goodfellow, M., and Bull, A.T. (2003). New primers for the class Actinobacteria: Application to marine and terrestrial environments. *Environ. Microbiol.* 5(10), 828-841. doi: 10.1046/j.1462-2920.2003.00483.x.

Nübel, U., Garcia-Pichel, F., and Muyzer, G. (1997). PCR primers to amplify 16S rRNA genes from cyanobacteria. *Appl. Environ. Microbiol.* 63(8), 3327-3332.

Manz, W., Amann, R., Ludwig, W., Vancanneyt, M., and Schleifer, K.-H. (1996). Application of a suite of 16S rRNA-specific oligonucleotide probes designed to investigate bacteria of the phylum cytophaga-flavobacter-bacteroides in the natural environment. *Microbiology* 142(5)**,** 1097-1106. doi: doi:10.1099/13500872-142-5-1097.

Muyzer, G., De Waal, E.C., and Uitterlinden, A.G. (1993). Profiling of complex microbial populations by denaturing gradient gel electrophoresis analysis of polymerase chain reaction-amplified genes coding for 16S rRNA. *Appl. Environ. Microbiol.* 59(3), 695-700.

Bacchetti De Gregoris, T., Aldred, N., Clare, A.S., and Burgess, J.G. (2011). Improvement of phylum- and class-specific primers for real-time PCR quantification of bacterial taxa. *J. Microbiol. Methods* 86(3)**,** 351-356. doi: http://dx.doi.org/10.1016/j.mimet.2011.06.010.
