# Supplementary material for: Distinct Seasonal Patterns of Bacterioplankton Abundance and Dominance of Phyla α-Proteobacteria and Cyanobacteria in Qinhuangdao Coastal Waters Off the Bohai Sea
Source: Front Microbiol. 2017 Aug 18;8:1579. doi: 10.3389/fmicb.2017.01579 (PMC5563310; doi:10.3389/fmicb.2017.01579)

Supplementary Material

Distinct seasonal patterns of bacterioplankton abundance and dominance of phyla α-Proteobacteria and Cyanobacteria in Qinhuangdao coastal waters off the Bohai Sea

Yaodong He[1, †], Biswarup Sen[1, †], Shuangyan Zhou[1], Ningdong Xie[1], Yongfeng Zhang[2], Jianle Zhang[2], Guangyi Wang[1, 3, *]

[†]These authors have contributed equally to this work.

[*]**Correspondence:** Corresponding Author: gywang@tju.edu.cn

Supplementary Figure S1: Classification and percentage distribution of the 16S rRNA gene sequences at family level. The bacterioplankton 16S rRNA gene sequence distribution in the six stations near the Qinhuangdao coastal area showed dominance of *Rhodobacteraceae* in station W2 (72%), W3 (42%), W5 (55%), and W6 (58%), and of *Family II* in station W1 (50%) and W4 (62%).

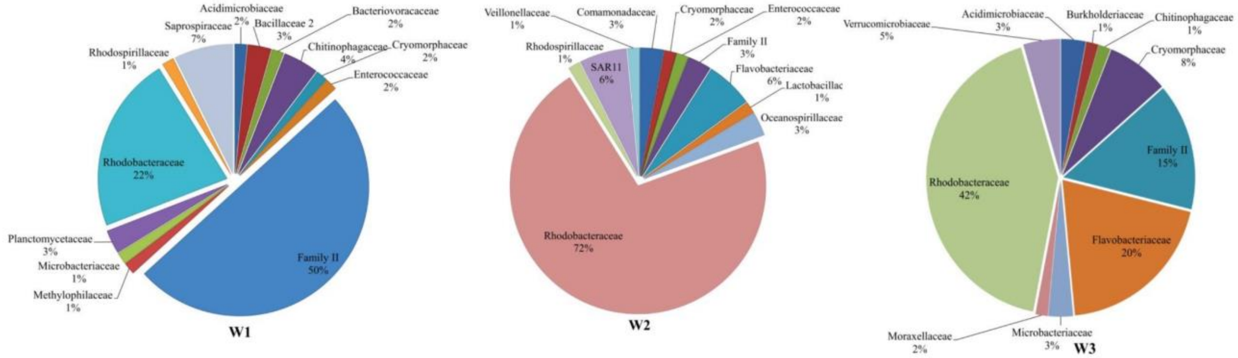

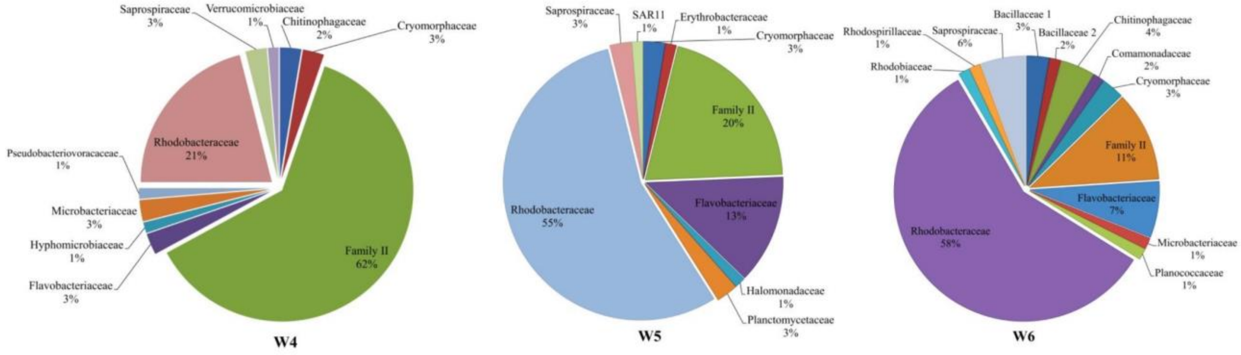

Supplement: Supplementary file 2 [file Image_1.pdf]
